# Supplementary material for: MetaRibo-Seq measures translation in microbiomes
Source: Nat Commun. 2020 Jun 29;11:3268. doi: 10.1038/s41467-020-17081-z (PMC7324362; doi:10.1038/s41467-020-17081-z)
Supplement: Supplementary file 10 — Supplementary Data 7 [file 41467_2020_17081_MOESM10_ESM.zip › File2/Confidence_High_Taxonomy/59256_out.krona.html]

Javascript must be enabled to view this page.

members
magnitude
magnitudeUnassigned
count
unassigned
taxon
rank

59256\_out

6

2759
4
superkingdom

kingdom

SRS051031\_contig\_number\_contig-100\_245.199201
4
1
4751

3
451864
subkingdom

3
4890
phylum

subphylum
3
147538

class
3
147548

order
5178
3

family
3
110844

3
2211661
genus

3
1436886
species

SRS013687\_contig\_number\_30894SRS023914\_contig\_number\_contig-100\_326.160672SRS1041145\_contig\_number\_31323

2
2
superkingdom

phylum

SRS053356\_contig\_number\_24330SRS076929\_contig\_number\_26875
2
1798710
